# Supplementary material for: Prohibitin 1 is essential to preserve mitochondria and myelin integrity in Schwann cells
Source: Nat Commun. 2021 Jun 2;12:3285. doi: 10.1038/s41467-021-23552-8 (PMC8172551; doi:10.1038/s41467-021-23552-8)
Supplement: Supplementary file 1 — Supplementary Information [file 41467_2021_23552_MOESM1_ESM.pdf]

Supplementary Information File for:

Prohibitin 1 is essential to preserve mitochondria and myelin integrity in Schwann cells.

Gustavo Della-Flora Nunes[1,2], Emma R. Wilson[1,2], Leandro N. Marziali[1,2], Edward Hurley[1], Nicholas Silvestri[3], Bin He[5], Bert W. O'Malley[6], Bogdan Beirowski[1,2], Yannick Poitelon[4], Lawrence Wrabetz[1,2,3], M. Laura Feltri[1,2,3]

[1]Hunter James Kelly Research Institute, Departments of [2]Biochemistry and [3]Neurology, Jacobs School of Medicine and Biomedical Sciences, University at Buffalo, Buffalo, NY 14203
[4]Albany Medical College, Dept of Neuroscience and Experimental Therapeutics, Albany, NY 12208
[5]Immunobiology & Transplant Science Center and Department of Surgery, Houston Methodist Hospital, Houston, TX, 77030
[6]Departments of Medicine and [6]Molecular and Cellular Biology, Baylor College of Medicine, Houston, TX, 77030

Supplementary Methods

Proliferation, TUNEL, SC numbers and macrophage staining

For proliferation and TUNEL assays, sciatic nerves were dissected, fixed in 4% PFA for 30 min, cryoprotected in sucrose and embedded in OCT tissue freezing medium. Longitudinal sections 8 μm thick were then obtained using a cryostat. For TUNEL assay, sections were permeabilized for 1 min using cold methanol, and sequentially incubated in TdT Buffer (30 mM Tris, 140 mM sodium cacodylate, 1 mM cobalt chloride) for 15 min at room temperature (RT), TdT enzyme mixture [60 U/ml Terminal Deoxynucleotidyl Transferase (TdT; Sigma-Aldrich), 6.25 mM Biotin-16-dUTP (Roche) in TdT buffer] for 1h at 37 ℃, TB buffer (300mM Sodium chloride, 40mM sodium citrate) for 15 min at RT, 2% BSA for 1h at RT and Alexa 594-Streptavidin secondary antibody (Jackson ImmunoResearch #016-580-084). Slides were then washed and counterstained with DAPI. For quantification of TUNEL and p-H3, 4-8 random areas (180 μm X 380 μm) were selected in each nerve and TUNEL+ and p-H3+ cells were counted and normalized to DAPI counts. For quantification of Ki-67, we co-stained SCs using SOX10 and calculated the percentage of Ki-67+ among SOX10+ cells from the entire nerve. Antibodies used were rabbit anti-p-H3 1/400 (Millipore #06-576) and rat anti-Ki-67 1/1000 (Thermo Fisher Scientific #14-5698-80). For quantification of SC number, nerves were prepared in the same way as for TUNEL/proliferation, but, in addition to longitudinal sections, we also sliced additional tissue in cross sections. Primary antibody was rabbit anti-SOX10 1/500 (Cell signaling #89356). For staining of macrophages, sciatic nerves were dissected and immediately embedded in OCT tissue freezing medium without fixation. Longitudinal sections 8 μm thick were then obtained using a cryostat. Primary antibody was rat anti-F4/80 (Biorad # MCA497GA). Staining for F4/80 required no permeabilization. For all the experiments mentioned above, images were acquired using the Zeiss ApoTome microscope (Zeiss Observer.Z1 AX10) running the AxioVision 4.8.2.0 software (Zeiss).

Mitophagy assessment

To detect mitophagy, primary rat SCs were transduced with a retroviral vector delivering a mitochondrially-targeted version of the pH sensitive protein mKeima [2]. The pCHAC-mt-mKeima was a gift from Richard Youle (Addgene plasmid # 72342). For the production of the retrovirus, we used the Retro-X Universal Packaging System (Takara #631530). Briefly, GP2-293 cells in a 10mm dish were transfected with 10 μg of pVSV-G envelope plasmid and 10 μg of pCHAC-mt-mKeima plasmid using calcium phosphate. Culture supernatant was collected 48h and 72h after transfection, filtered with a 0.22 μm filter, ultra-centrifuged at ~70,000 g for 2h at 4°C and the pellet was resuspended in PBS. Culture media of GP2-293 cells was DMEM supplemented with 10% FBS, 4mM/L L-glutamine, 100 U/mL penicillin, 100 μg/mL streptomycin and 1mM sodium pyruvate. After transduction with the retrovirus, we passaged the primary rat SCs cells and allowed them to recover for 24h. We then knocked down *Phb1* using shRNA delivered through a lentivirus (described in "Cell culture and *Phb1* knockdown" sub-section). 48h after knockdown of *Phb1*, mitophagy was induced in SCs using a combination of 10 μM FCCP (Sigma # C2920) and 1 μM oligomycin (EMD Millipore # 495455). Cells were live imaged 24h later. Mitochondrial mKeima was detected by stimulating cells with the 458nm laser, while mitochondria undergoing degradation in lysosomes was detected by stimulation with the 594nm laser. Each data point represents the average for 2-5 cells in each well. Data were collected from two independent experiments. Quantifications were performed using auto-thresholding in ImageJ Fiji v1.52p.

Thiobarbituric acid reactive substances (TBARS) assay

Lipoperoxidation was evaluated using a TBARS assay kit (Abnova #KA1381), according to the manufacturer's instructions. Sciatic nerves were weighed (approximately 25mg), pulverized in liquid nitrogen and resuspended in the same lysis buffer used for western blot. Lysates were centrifuged at 13,200 g for 15min at 4°C and supernatant was used in the assay. Results were detected using fluorescence (excitation 530 nm, emission 550 nm, bandwidth 9-nm) in the Biotek Cytation 5 plate reader. Malondialdehyde (MDA) concentration was estimated by comparison to a standard curve and results were normalized by protein concentration determined using a BCA protein assay (Thermo Scientific).

Protein carbonyl content

Protein oxidation was evaluated using a protein carbonyl assay kit (Cayman chemical #10005020), according to the manufacturer's instructions. Briefly, sciatic nerves were dissected, stripped from epineurium and other contaminant tissue and snap frozen in liquid nitrogen until the analysis. Nerves were pulverized in liquid nitrogen and resuspended in the same lysis buffer used for western blot. Lysates were centrifuged at 13,200 g for 15min at 4°C and supernatants were polled two by two (respecting animal genotypes) to create the samples used in the assay. Protein concentration was determined at this stage using a BCA protein assay (Thermo Scientific). Protein carbonyl content was detected using colorimetry (370 nm) in the Biotek Cytation 5 plate reader. Protein carbonyl concentration was calculated following the kit's instructions and results were normalized by the previously assessed protein concentration.

**Supplementary Table 1. List of primers**				
**Gene**	**Forward primer (3'-5')**	**Reverse primer (3'-5')**	**Probe (if applicable)**	**Reference**
Phb1	gggtcctgccttctatcacc	tcaattctccagcatcgaatc	UPL #77	
Phb2	ccattgttaatgaggtgccaa	cttcggatcaacagggaca	UPL #49	
Ddit3	accaccacacctgaaagca	gacctcctgcagatcctcat	UPL #11	
Atf4	tataaagggcgggtttaggg	acttaaaccggcagacagca	UPL #70	
Clpp	gagctttcccgctcatcc	ggccacactgtcgtcaatc	UPL #22	
Asns	ggccacactgtcgtcaatc	aggaaggaagggctccact	UPL #22	
Chac1	gtatcacctgcccatgttcc	aagagctacttcgcctccttc	UPL #56	
Pck2	ggcagagcacatgctgatt	gccacgtagcgctttttc	UPL #9	
Psph	agcctgtcctcctgctgat	gtaccacttggcgttgtcct	UPL #62	
Hspd1	cagtggaaatccggagagg	aggtttagactgtttcttaagttcagc	UPL #89	
Hspe1	ggcccgagttcagagtcc	ctgtcaaagagcggaagaaac	UPL #62	
Mcp1	catccacgtgttggctca	gatcatcttgctggtgaatgagt	UPL #56	
Actb (β-actin)	aaggccaaccgtgaaaagat	gtggtacgaccagaggcatac	UPL #56	
Srebp1	catgccatgggcaagtacac	tgttgccatggagatagcatct		[3]
Hgmcr	tggtgggaccaaccttctac	gccatcacagtgccacatac		[3]
Acly	aggaagtgccacctccaacagt	cgctcatcacagatgctggtca		[3]
Fasn	gggtgctgactacaacctctcc	tgcacagacaccttcccgtc		[3]
Acc2	gggctccctggatgacaac	tcttccgggaggagttct		[3]
mitochondrial DNA	aagtcgtaacaaggtaagca	atatttgtgtagggctaggg		[3]
nuclear DNA	gggtatatttttgataccttcaatgagtta	tctgaaacagtaggtagagaccaagac		[3]
mt-Co1	gcaggagcatcagtagacctaac	ggagtttgatactgtgttatggctgg		[4]
mt-Nd1	cacctaccctatcactcacactagc	ggctcatcctgatcatagaatggag		[4]
mt-Atp6	ccttccacaaggaactccaatttcac	ctagagtagctcctccgattaggtg		[4]
Tfam	caggaggcaaaggatgattc	atgtctccggatcgtttcac		[5]
Sdhb	tgtagagaaggcatctgtgg	cgtagaagttactcaaatcaggg		[5]
Pgc1a	gaatcaagccactacagacaccg	catccctcttgagcctttcgtg		[6]

Supplementary Table 1. List of primers used in this study.

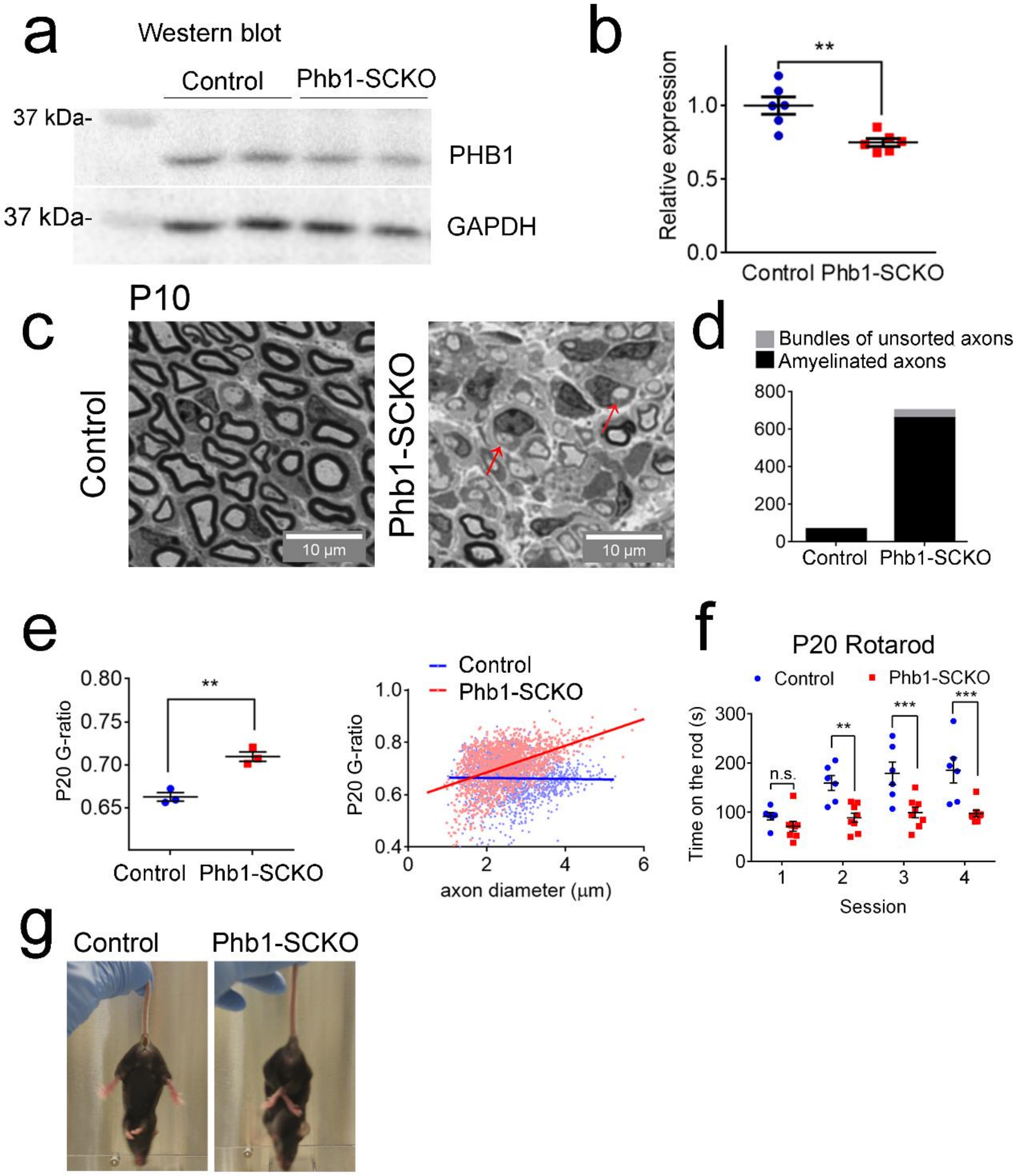

Supplementary Fig. 1. Phb1-SCKO animals show a minor developmental delay in myelination. (**a**) Recombination of the floxed *Phb1* gene leads to a reduced PHB1 protein levels in sciatic nerve lysates of postnatal day 20 (P20) PHB1-SCKO mice. (**b**) Quantification of the PHB1 western blot. N=6 animals per genotype. Unpaired two-tailed t-test (t=3.888, df=10, p=0.003). (**c**) Semithin cross sections of sciatic nerves of 10-day-old mice. Arrows indicate pro-myelinating SCs (in a one-to-one relationship with unmyelinated axons) (**d**) Quantification reveals an increased amount of pro-myelinating SCs in Phb1-SCKO animals when compared to controls. Average of 3-4 animals per genotype. (**e**) By P20, the majority of axons larger than 1 μm in diameter have been myelinated, but myelin thickness is reduced (increased g-ratio = the ratio between axon and fiber diameters), especially in axons of larger caliber. N=3 animals per genotype. Unpaired two-tailed t-test (t=6.306, df=4, p=0.0032). (**f**) At P20, Phb1-SCKO mice also show an impaired performance on the accelerating rotarod after session 2. N=6-7 animals per genotype. Repeated measures one-way ANOVA corrected for multiple comparisons using the Holm-Sidak method. F (1,12) group = 21.28, p<0.01; F (3,36) time = 11.78, p<0.001; F (3,36) interaction = 3.524, p<0.05; p_{day1}=0.30; p_{day2}= 0.0017; p_{day3}= 0.0006; p_{day4}= 0.0002. (**g**) Representative images of the clenching phenotype in P90 Phb1-SCKO mice. Data are presented as mean +/- SEM. ** p<0.01; *** p<0.001. n.s. = non-significant.

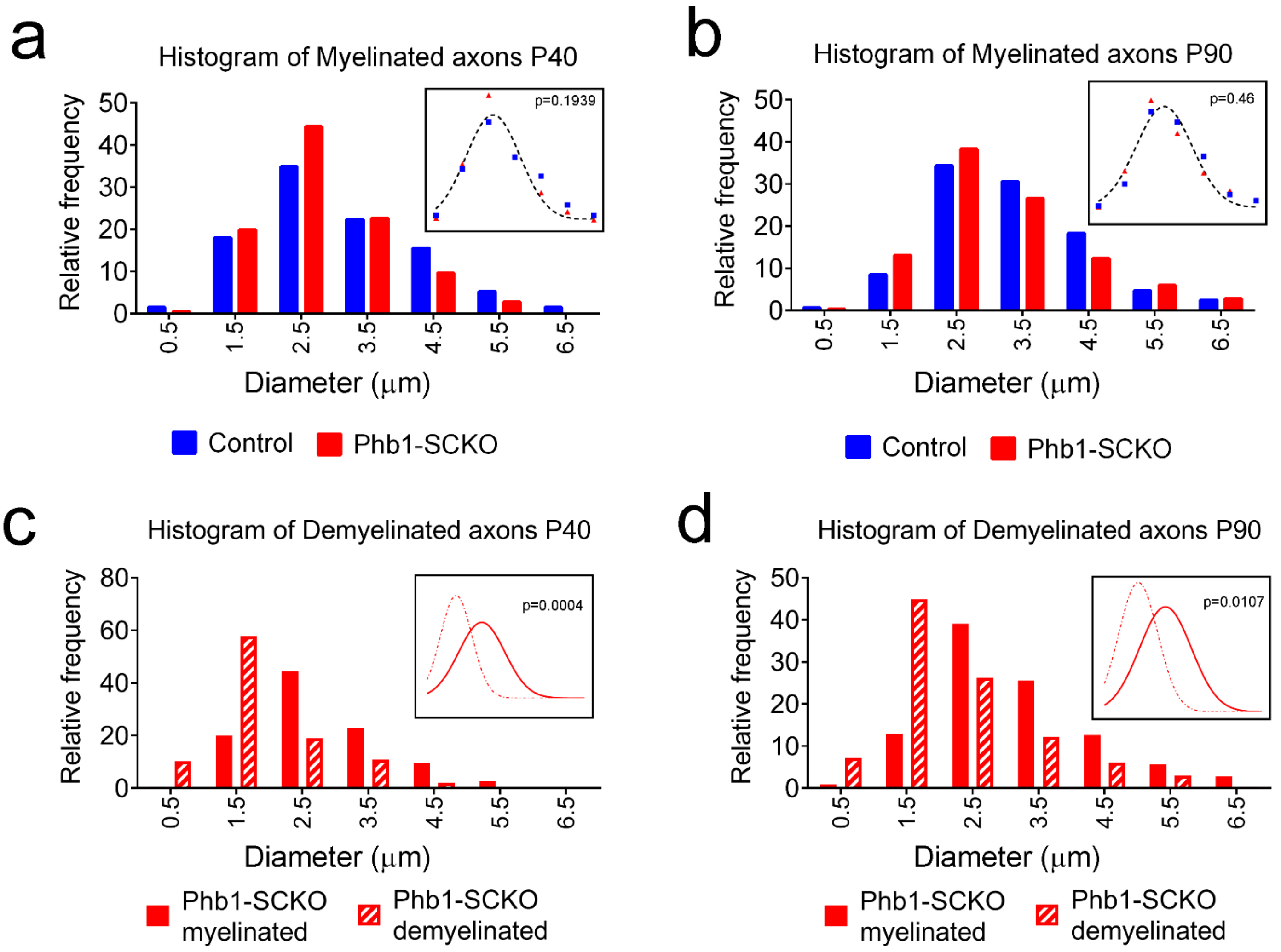

Supplementary Fig. 2. Size distribution of myelinated axons in sciatic nerves is preserved in Phb1-SCKO mice at postnatal day 40 (P40) (**a**) and P90 (**b**). However, demyelinated axons show a shift toward smaller caliber in 40 (**c**) and 90-day-old Phb1-SCKO mice (**d**). N=3 animals per genotype. Insets: nonlinear regression using a Gaussian curve followed by extra sum-of-squares F test [F(3,8) myelinated axons P40=1.992, F(3,8) myelinated axons P90=0.9529, F(3,8) demyelinated axons P40=20.05, F(3,8) demyelinated axons P90=7.406].

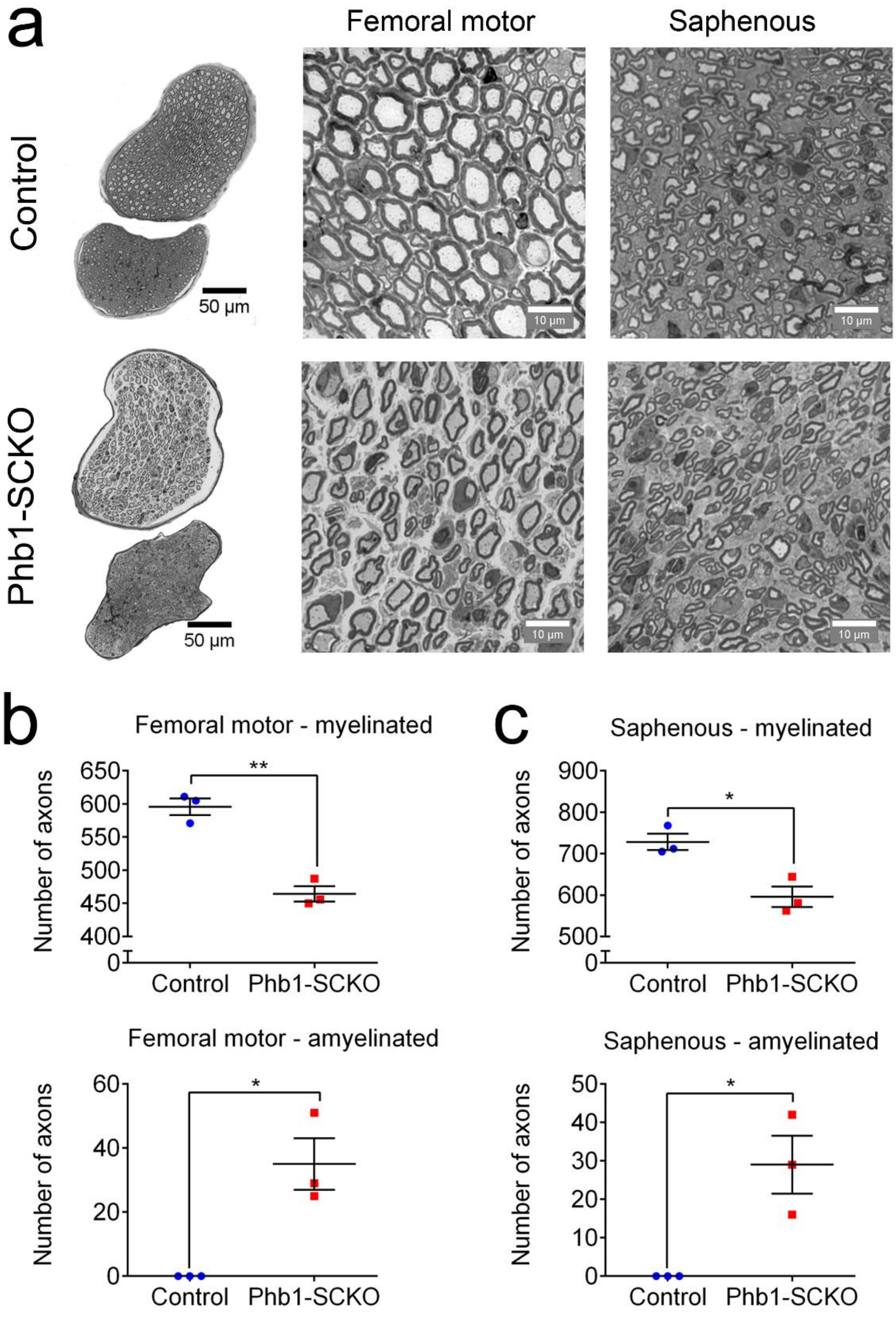

Supplementary Fig. 3. The neuropathy caused by Phb1 ablation in SCs affects both motor and sensory fibers. (**a**) Representative images of the femoral nerve branches in semithin cross sections stained with toluidine blue. In 40-day-old Phb1-SCKO animals, both the femoral motor branch (**b**) and the saphenous sensory branch (**c**) show a significant reduction in number of myelinated fibers and an increase in number of amyelinated axons. N=3 animals per genotype. Unpaired two-tailed t-test. Femoral motor [myelinated (t=7.758, df=4, p=0.0015), amyelinated (t=4.330, df=4, p=0.0123)]; Saphenous [myelinated (t=4.171, df=4,p= 0.014), amyelinated (t=3.864, df=4, p=0.018)]. Data are presented as mean +/- SEM. * p<0.05; ** p<0.01.

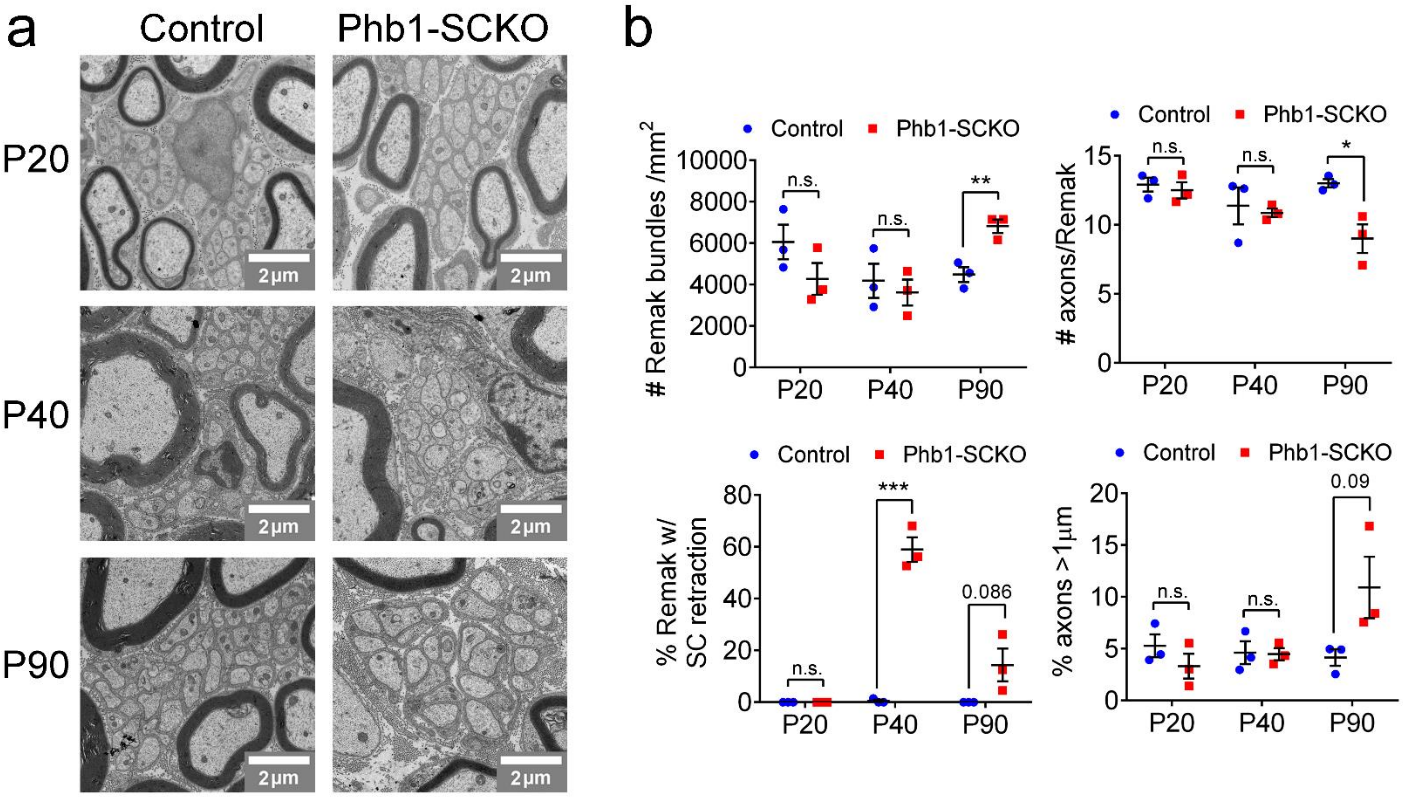

Supplementary Fig. 4. Remak bundles are progressively affected by ablation of *Phb1*. (**a**) Representative electron micrographs of Remak bundles in sciatic nerves of Phb1-SCKO and controls at postnatal day 20 (P20), P40 and P90. (**b**). Remak bundles of 20-day-old Phb1-SCKO mice (red) do not show any abnormalities and are comparable to controls (blue) in number of Remak bundles per area, number of axons per Remak bundle and percentage of axons larger than 1 μm in diameter. However, Remak SCs of P40 Phb1-SCKO animals appear to have retracted from the axons and, at P90, there is an increased number of Remak bundles per area and a reduced number of axons per Remak bundle. N=3 animals per genotype. Unpaired two-tailed t-test. # Remak bundles [P20 (t=1.577, df=4, p=0.19), P40 (t=0.5424, df=4, p=0.62), P90 (t=4.779, df=4, p=0.0088)]; # axons/ Remak [P20 (t=0.5317, df=4, p=0.62), P40 (t=0.3692, df=4, p=0.73), P90 (t=3.722, df=4, p=0.02)]; % Remak w/ SC retraction [P40 (t=12.34, df=4, p=0.00025), P90 (t=2.273, df=4, p=0.086)]; % axons > 1μm [P20 (t=1.211, df=4, p=0.29), P40 (t=0.1068, df=4, p=0.92), P90 (t=2.206, df=4, p=0.092)]. Data are presented as mean +/- SEM. * p<0.05; ** p<0.01; *** p<0.001. n.s. = non-significant.

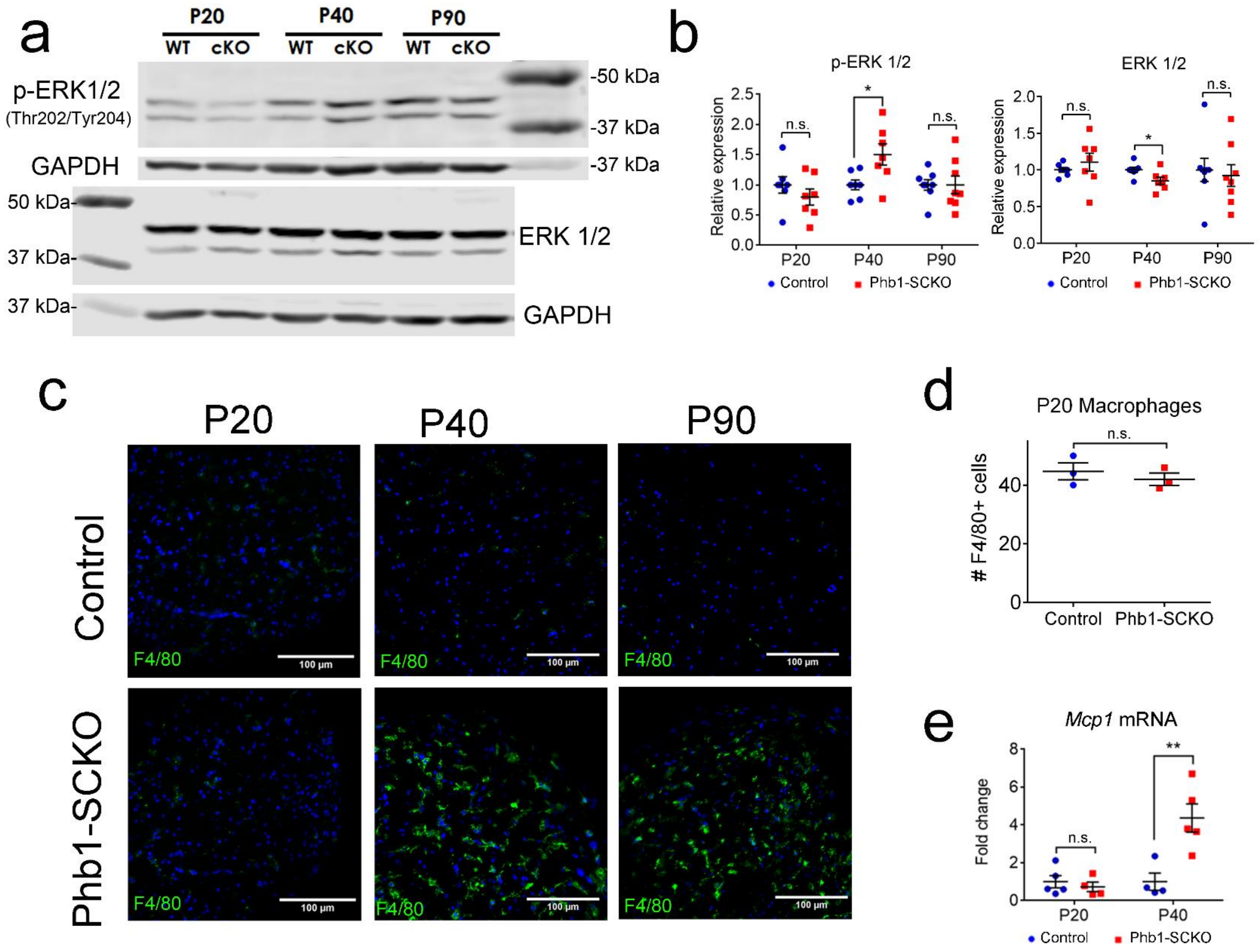

Supplementary Fig. 5. Macrophage infiltration is a late event and may happen downstream of ERK signaling. (**a**) There are only temporary changes in the ERK signaling pathway in Phb1-SCKO mice. (**b**) Quantification of (a). N=6-8 animals per genotype. Unpaired two-tailed t-test. p-ERK 1/2 [P20 (t=1.048, df=12, p=0.32), P40 (t=2.592, df=12, p=0.024), P90 (t=0.169, df=14, p=0.997)]; ERK 1/2 [P20 (t=0.831, df=12, p=0.42), P40 (t=2.454, df=12, p=0.03), P90 (t=0.361, df=14, p=0.72)]. (**c**) Abundant macrophage infiltration occurs in response to pathology in sciatic nerves of Phb1-SCKO mice. Macrophage infiltration (green) is noticeable starting at postnatal day 40 (P40) and is still present at P90 (middle and right panels). (**d**) There is no difference in the number of macrophages between genotypes in sciatic nerves of 20-day-old animals. N=3 animals per genotype. Unpaired two-tailed t-test (t=0.7460, df=4, p=0.5). (**e**) Expression of monocyte chemoattractant protein 1 (*Mcp1*) is also upregulated concomitantly with p-ERK elevation at P40. Unpaired two-tailed t-test [P20 (t=0.627, df=7, p=0.55), P40 (t=3.587, df=7, p=0.0089)]. Data are presented as mean +/- SEM. * $p<0.05$; ** $p<0.01$. n.s. = non-significant.

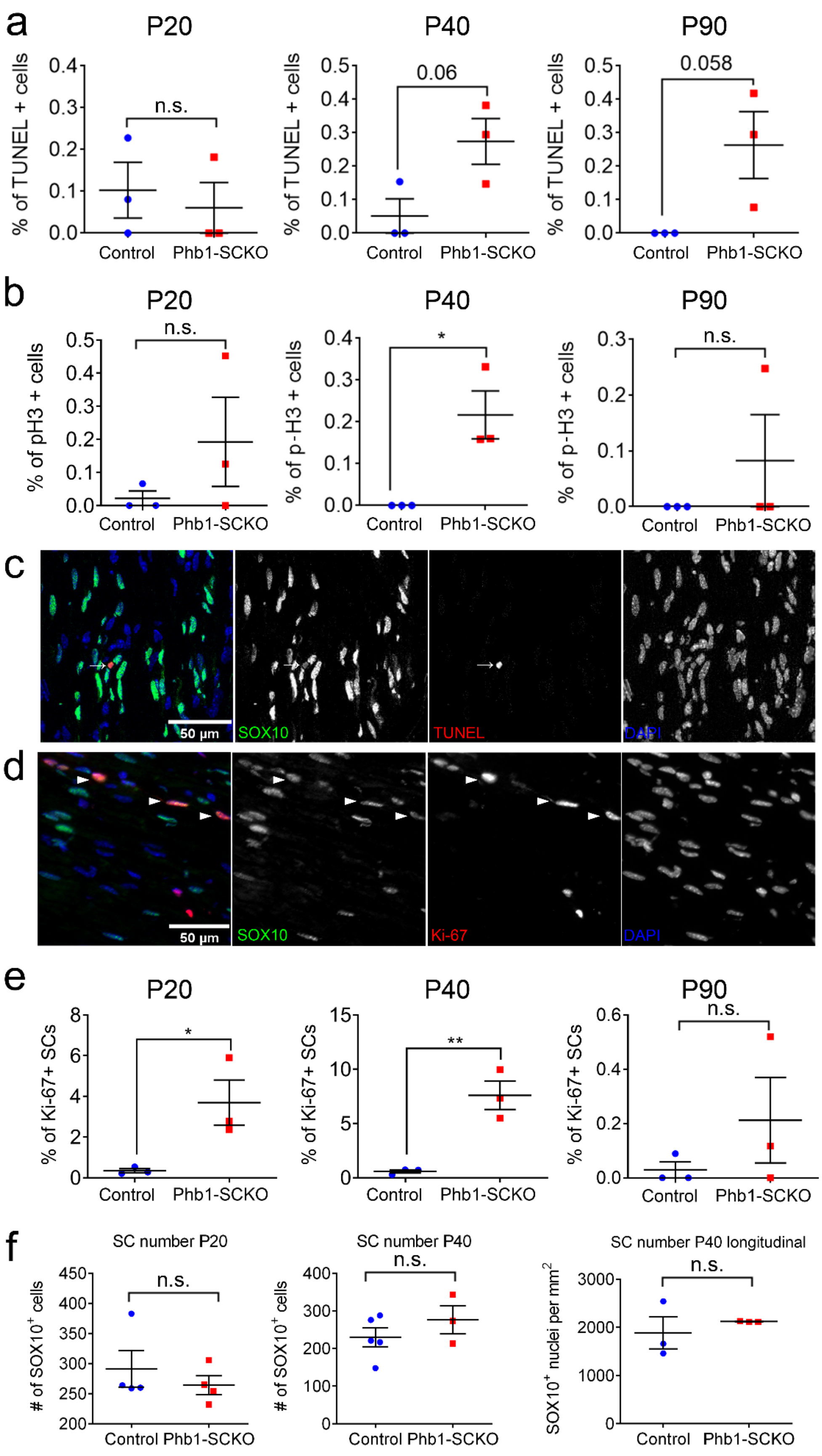

Supplementary Fig. 6. Schwann cell number is not altered by deletion of *Phb1* (**a-b**) Proliferation and apoptosis are slightly altered as a result of *Phb1* deletion in SCs. Neither TUNEL (cell death - **a**) or phospho-Histone 3 (p-H3; mitosis - **b**) staining is significantly changed in postnatal day 20 (P20) Phb1-SCKO animals (red) in comparison to littermate controls (blue; left panels). However, both measurements are slightly increased at P40 (middle and right panels). N=3 animals per genotype. Unpaired two-tailed t-test. TUNEL [P20 (t=0.4694, df=4, p=0.66), P40 (t=2.609, df=4, p=0.06), P90 (t=2.636, df=4, p=0.058)]; p-H3 [P20 (t=1.247, df=4, p=0.28), P40 (t=3.777, df=4, p=0.019), P90 (t=1.000, df=4, p=0.37)]. (**c**) Co-staining of TUNEL (red) and SOX10 (green) indicates that TUNEL+ cells are also SOX10+ (arrows). N=3 Phb1-SCKO animals. (**d**) Most proliferating cells (Ki-67+; red) are SCs (SOX10+; green) (arrowheads). N=3 Phb1-SCKO animals. (**e**) There is a significant increase in percentage or proliferating SCs (Ki-67+) in nerves of Phb1-SCKO mice at P20 and P40. N=3 animals per genotype. Unpaired two-tailed t-test. P20 (t=2.992, df=4, p=0.04), P40 (t=5.350, df=4, p=0.006), P90 (t=1.140, df=4, p=0.32)]. (**f**) Total number of nuclei stained with SOX10 in cross sections of sciatic nerves is similar in both genotypes at P20 and P40 (left and middle panels). N=3-5 animals per genotype. Unpaired two-tailed t-test. [P20 (t=0.7959, df=6, p=0.46), P40 (t=1.074, df=6, p=0.32). The same result is obtained at P40 when SOX10+ nuclei are counted in longitudinal sections of the sciatic nerve (right panel). N=3 animals per genotype. Unpaired two-tailed t-test (t=0.6909, df=2, p=0.56). Data are presented as mean +/- SEM. * p<0.05; ** p<0.01. n.s. = non-significant.

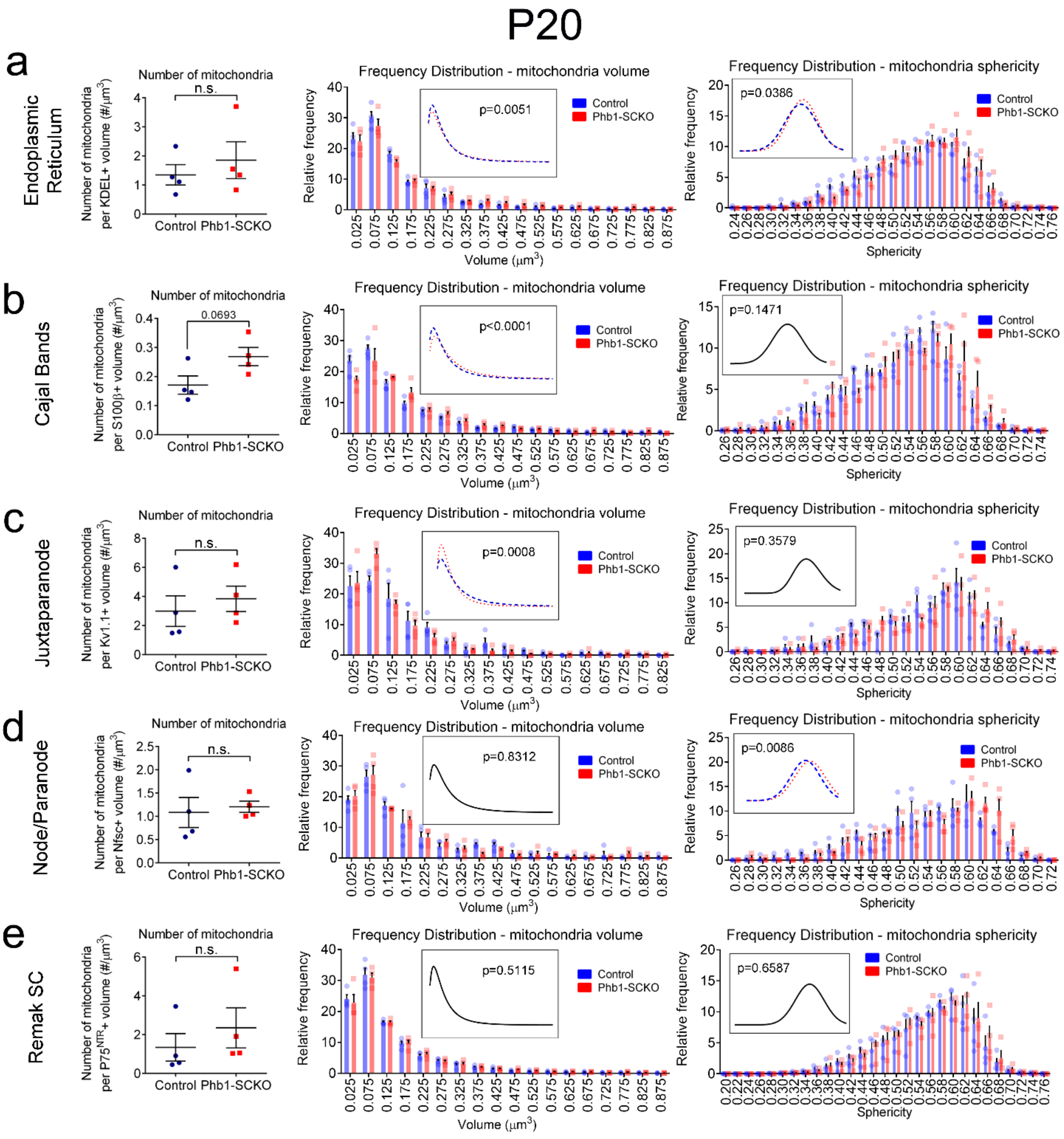

Supplementary Fig. 7. Early changes in mitochondrial morphology are detected using the PhAM reporter line in postnatal day 30 (P20) Phb1-SCKO mice. When lacking *Phb1*, mitochondria around the endoplasmic reticulum (**a**) and in Cajal bands (**b**) show a shift towards a larger size. (**c**) On the other hand, mitochondria in the juxtaparanodal region of Phb1-SCKO mice show a shift towards smaller size. There is only a small change in sphericity of mitochondria around the node/paranodes (**d**), and no significant changes in mitochondria in the Remak SCs (**e**). Figures show the average quantification for mitochondria in the indicated cell compartment. N = 4 animals per genotype. Unpaired two-tailed t-test was used to compare the mitochondrial numbers, while a nonlinear regression followed by an extra sum-of-squares F test was used to compare the distributions of volume and sphericity using a lognormal or Gaussian distribution, respectively. Number of mitochondria [ER (t=0.6980, df=6, p=0.51), Cajal bands (t=2.208, df=6, p=0.069), Juxtaparanode (t=0.6180, df=6, p=0.56), Node/Paranode (t=0.3493, df=6, p=0.74), Remak SC (t=0.7977, df=6, p=0.46)]. Volume [F (3,138) ER = 4.458, F (3,138) Cajal bands = 14.4, F (3,130) Juxtaparanode = 5.941, F (3,138) Node/Paranode = 0.2919, F (3,138) Remak SC = 0.7721]. Sphericity [F (3,210) ER = 2.846, F (3,194) Cajal bands = 1.808, F (3,194) Juxtaparanode = 1.082, F (3,186) Node/Paranode = 4.006, F (3,226) Remak SC = 0.5351]. Data are presented as mean +/- SEM. n.s. = non-significant.

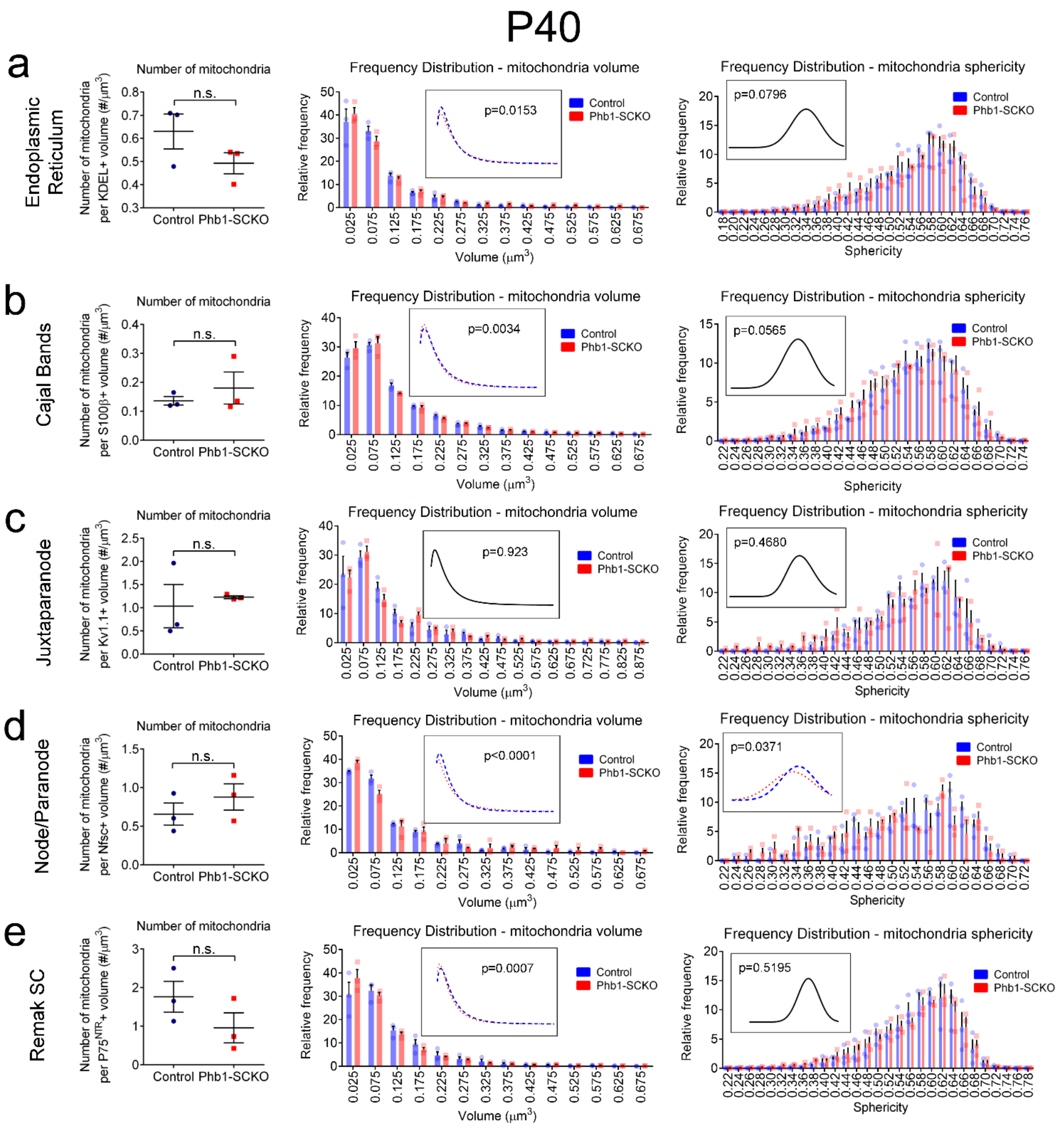

Supplementary Fig. 8. Additional morphological changes in mitochondria of postnatal day 40 (P40) Phb1-SCKO animals. When lacking *Phb1*, mitochondria around the endoplasmic reticulum (**a**) and around nodes/paranodes (**d**) show a shift towards a larger size. On the other hand, mitochondria in Cajal bands (**b**) and non-myelinating SCs (**e**) of Phb1-SCKO mice show a shift towards smaller size. There are no significant changes in mitochondria surrounding the juxtaparanodes (**d**). Figures show the average quantification for mitochondria in the indicated cell compartment. N= 3 animals per genotype. Unpaired two-tailed t-test was used to compare the mitochondrial numbers, while a nonlinear regression followed by an extra sum-of-squares F test was used to compare the distributions of volume and sphericity using a lognormal or Gaussian distribution, respectively. Number of mitochondria [ER (t=1.559, df=4, p=0.19), Cajal bands (t=0.7726, df=4, p=0.48), Juxtaparanode (t=0.4156, df=4, p=0.7), Node/Paranode (t=0.9947, df=4, p=0.38), Remak SC (t=1.437, df=4, p=0.22)]. Volume [F (3,78) ER = 3.693, F (3,78) Cajal bands = 4.938, F (3,102) Juxtaparanode = 1.16, F (3,78) Node/Paranode = 8.914, F (3,78) Remak SC = 6.356]. Sphericity [F (3,174) ER = 2.295, F (3,156) Cajal bands = 2.567, F (3,162) Juxtaparanode = 0.8509, F (3,150) Node/Paranode = 2.898, F (3,168) Remak SC = 0.7574]. Data are presented as mean +/- SEM. n.s. = non-significant.

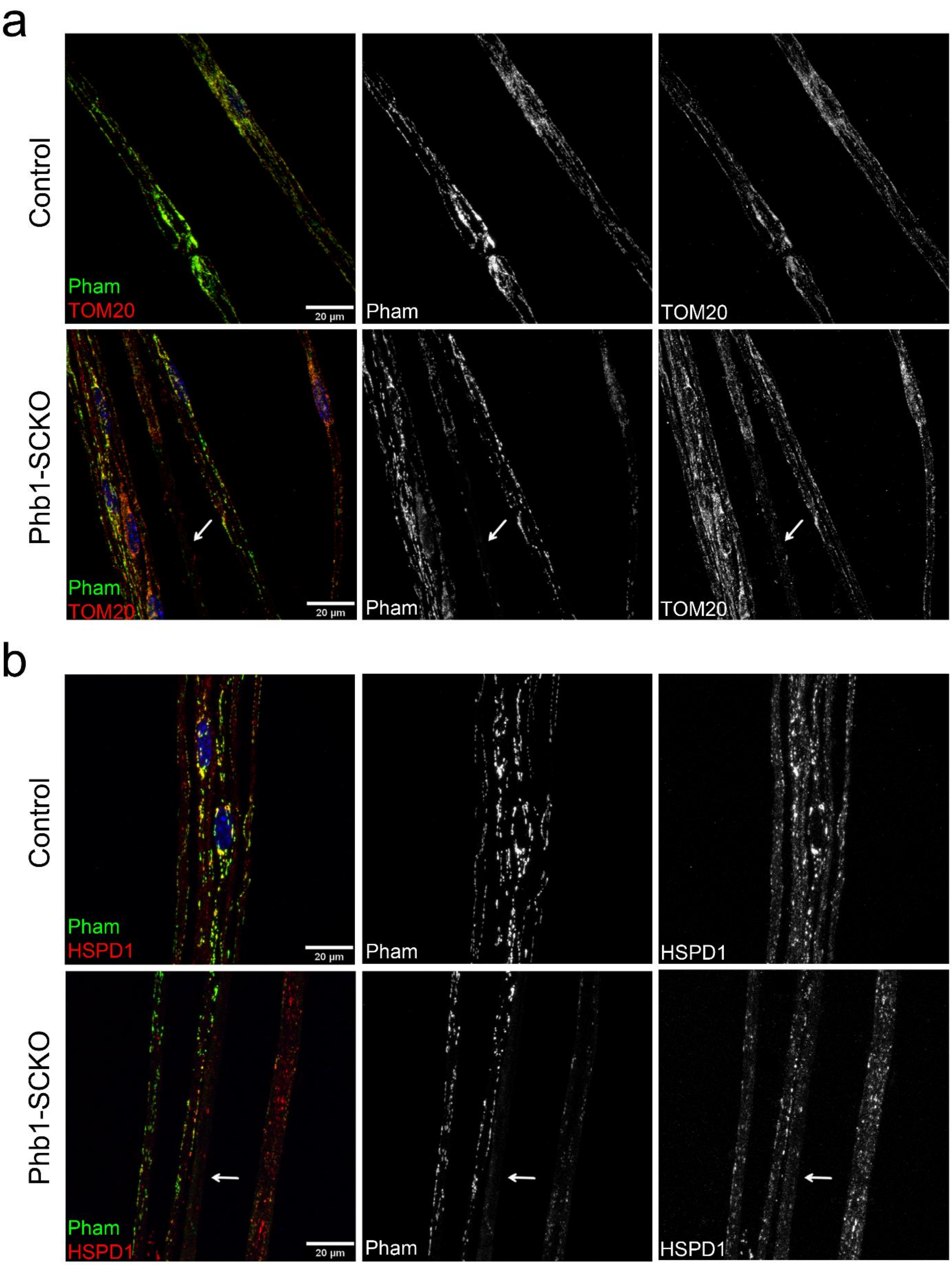

Supplementary Fig. 9. Different mitochondrial markers confirm the results obtained using the PhAM reporter in postnatal day 40 (P40) Phb1-SCKO mice. (**a**-**b**) SCs in which PhAM (green) is undetectable also show reduced staining for the mitochondrial proteins TOM20 (**a**) and HSPD1 (**b**), in red, suggesting severe damage and fragmentation of mitochondria. Arrows point to SCs with damaged mitochondria in Phb1-SCKO animals. N = 3 animals per genotype per condition.

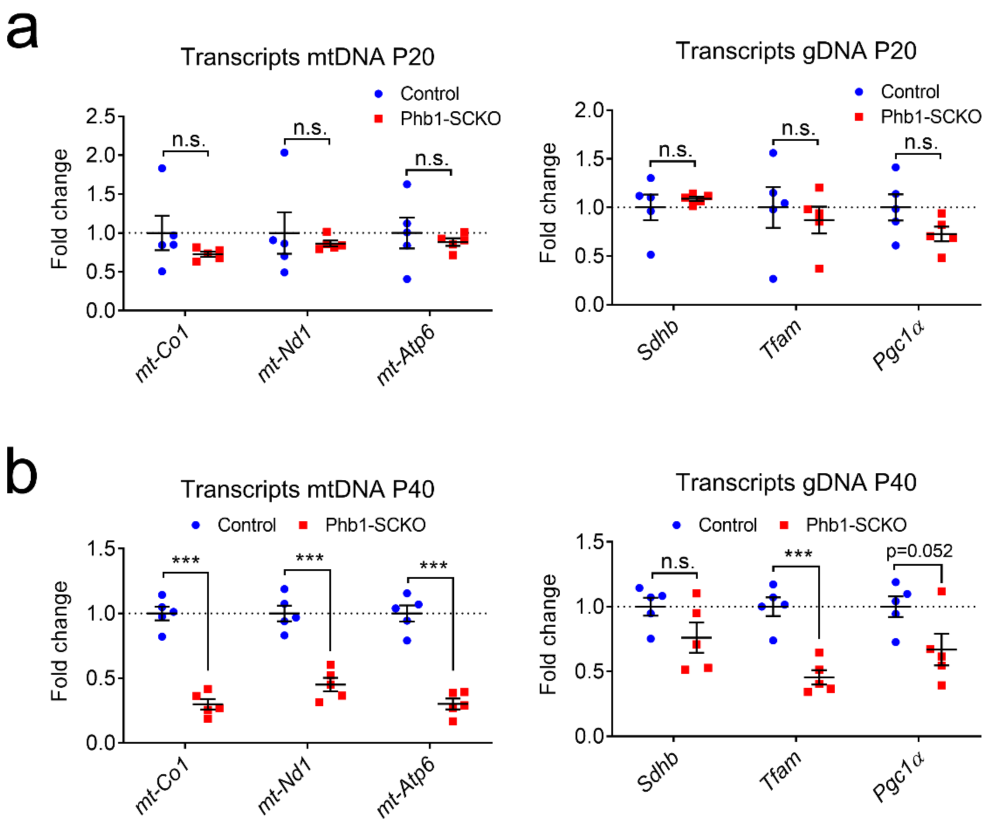

Supplementary Fig. 10. Transcriptional regulation of mitochondrial genes is impaired by deletion of *Phb1* in SCs. (**a**) There are no changes in mRNA levels of transcripts from the mitochondrial DNA (mtDNA) or genomic DNA (gDNA) at postnatal day 20 (P20). N = 5 animals per genotype. Unpaired two-tailed t-test. mtDNA P40: *mt-Co1* (t=1.21, df=8, p=0.26), *mt-Nd1* (t=0.5, df=8, p=0.63), *mt-Atp6* (t=0.57, df=8, p=0.58); gDNA P40: *Sdhb* (t=0.65, df=8, p=0.53), *Tfam* (t=0.52, df=8, p=0.62), *Pgc1α* (t=1.76, df=8, p=0.12). (**b**) At P40, there is a significant reduction of transcripts encoded in the mtDNA and of transcription factor A mitochondrial (*Tfam*) and peroxisome proliferator-activated receptor gamma coactivator 1-alpha (*Pgc1α*), key transcriptional regulators in the mitochondria. However, there is no change in expression level of succinate dehydrogenase complex subunit B (*Sdhb*), which codes for a subunit of mitochondrial complex II, the only mitochondrial complex encoded exclusively in the gDNA. N = 5 animals per genotype. Unpaired two-tailed t-test. mtDNA P40: *mt-Co1* (t=10.55, df=8, p=0.000006), *mt-Nd1* (t=6.85, df=8, p=0.000131), *mt-Atp6* (t=9.31, df=8, p=0.000014); gDNA P40: *Sdhb* (t=1.76, df=8, p=0.116188), *Tfam* (t=5.99, df=8, p=0.000328), *Pgc1α* (t=2.29, df=8, p=0.051631). Data are presented as mean +/- SEM. *** p<0.001. n.s. = non-significant.

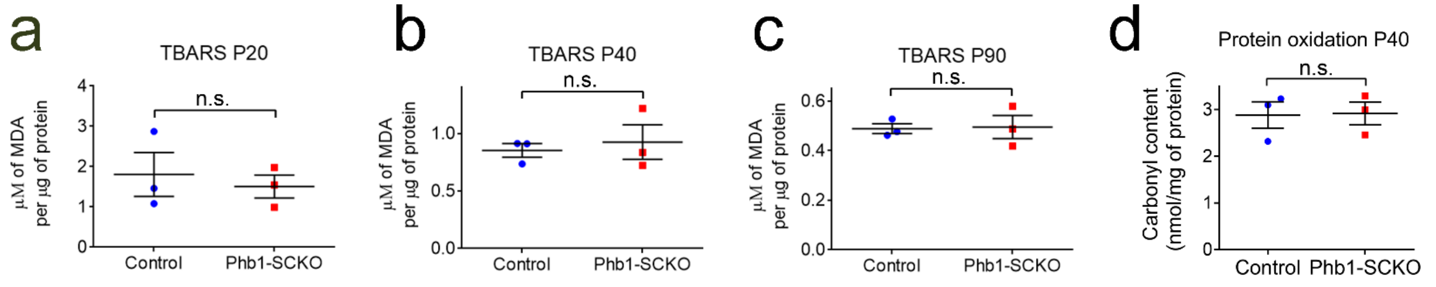

Supplementary Fig. 11. There is no evidence for presence of oxidative damage in nerves of Phb1-SCKO mice. (**a-c**) Thiobarbituric acid reactive substances (TBARS) assay to test for lipoperoxidation in sciatic nerve lysates. N= 3 animals per genotype. Unpaired two-tailed t-test [P20 (t=0.4871, df=4, p=0.65), P40 (t=0.4484, df=4, p=0.68), P90 (t=0.1253, df=4, p=0.91)]. (**d**) Protein carbonyl content in sciatic nerve lysates. N=3 samples per genotype pooled from 2 animals each. Unpaired two-tailed t-test (t=0.08650, df=4, p=0.94). Data are presented as mean +/- SEM. n.s. = non-significant.

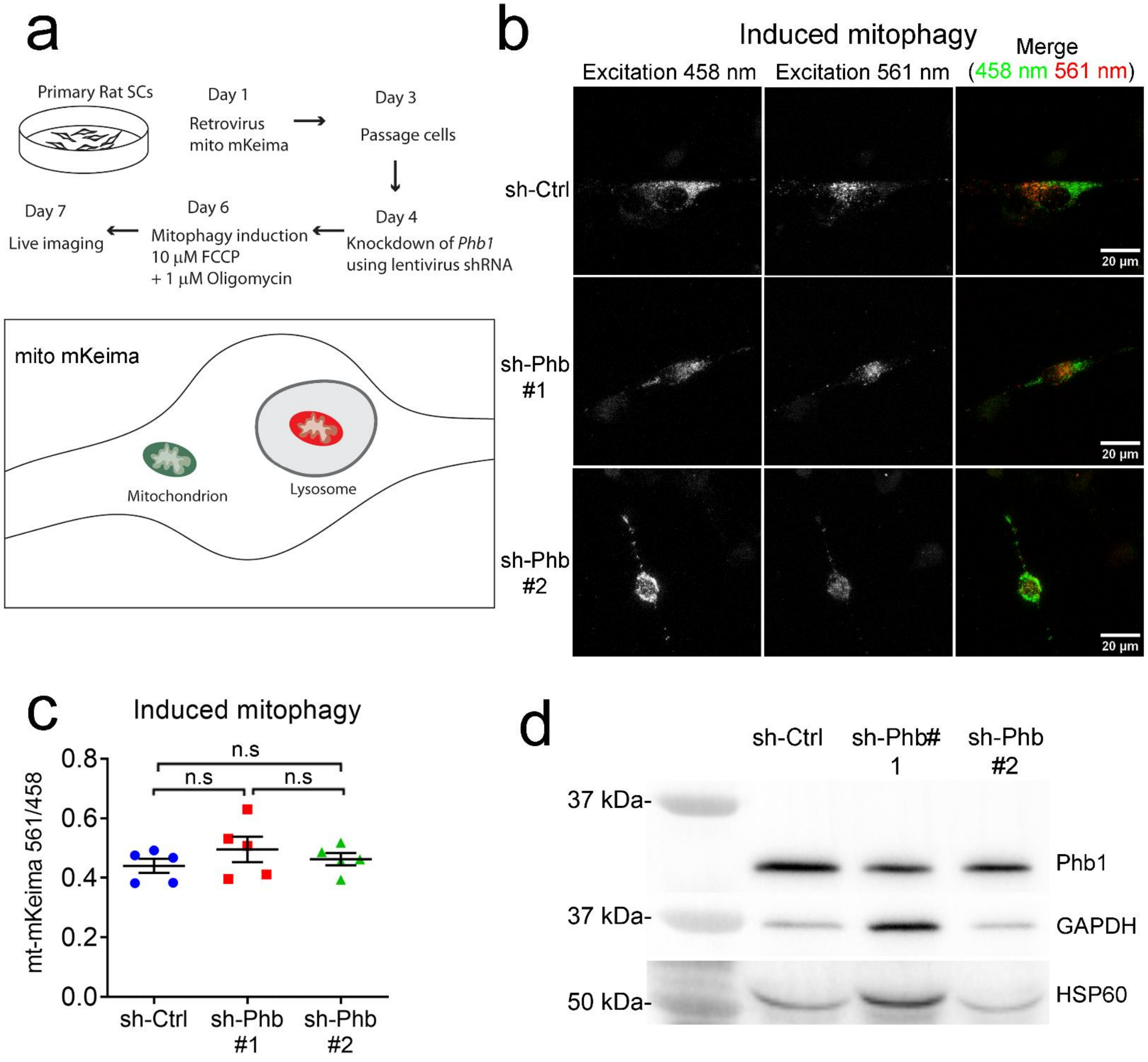

Supplementary Fig. 12. The capacity of Schwann cells to perform mitophagy is not affected by reduction in the expression of *Phb1*. (**a**) Schematic representation of the experiment. mito mKeima is a pH sensitive fluorescent protein that switches the excitation spectrum from “green” to “red” when mitochondria undergo degradation in lysosomes. (**b**) Representative immunofluorescence images of cells of each condition. (**c**) Quantification of (b). There is no difference between groups. N=5 wells per condition. one-way ANOVA corrected for multiple comparisons using the Holm-Sidak method. F (2,12) group = 0.8213, p=0.4631. (**d**) Western blot illustrating the reduction in levels of PHB1 upon treatment with shRNAs (65% and 56% reduction in PHB1/HSP60 ratio for sh-Phb #1 and sh-Phb #2, respectively). Experiment was repeated independently twice. Data are presented as mean +/- SEM. n.s. = non-significant.

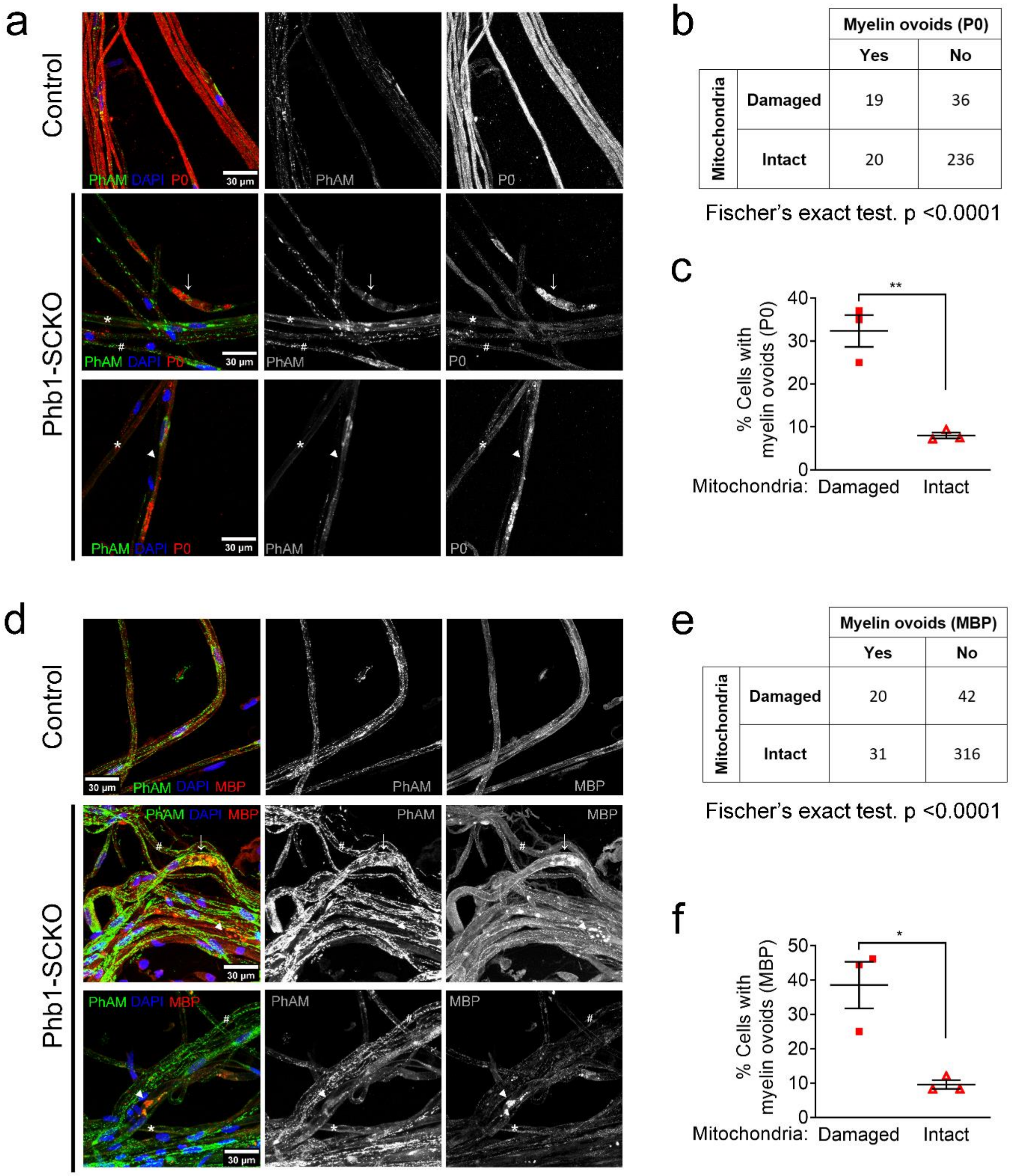

Supplementary Fig. 13. Mitochondrial damage is linked to demyelination. (**a**) Immunofluorescence of teased fibers of postnatal day 40 (P40) control and Phb1-SCKO mice expressing the PhAM mitochondrial reporter (green) and immunostained for myelin protein zero (P0; red). (**b**) Quantification of each subcategory of cell in (a). N= 3 animals. Two-tailed Fischer's exact test. (**c**) A greater proportion of cells with damaged mitochondrial network present with P0+ myelin ovoids. N= 3 animals. Unpaired two-tailed t-test (t=6.434, df=4, p=0.003). (**d**) Immunofluorescence of teased fibers of P40 control and Phb1-SCKO mice expressing the PhAM mitochondrial reporter (green) and immunostained for myelin basic protein (MBP; red). (**e**) Quantification of each subcategory of cell in (d). N= 3 animals. Two-tailed Fischer's exact test. (**f**) A greater proportion of cells with damaged mitochondrial network present with MBP+ myelin ovoids N= 3 animals. Unpaired two-tailed t-test (t=4.200, df=4, p=0.014). Arrowhead: cells with myelin ovoids and damaged mitochondria. Arrow: cells with myelin ovoids, but relatively intact mitochondria. Star: Cells with damaged mitochondria, but no myelin ovoids. Pound: cells with intact mitochondria and no myelin ovoids. Data are presented as mean +/- SEM. * p<0.05; ** p<0.01.

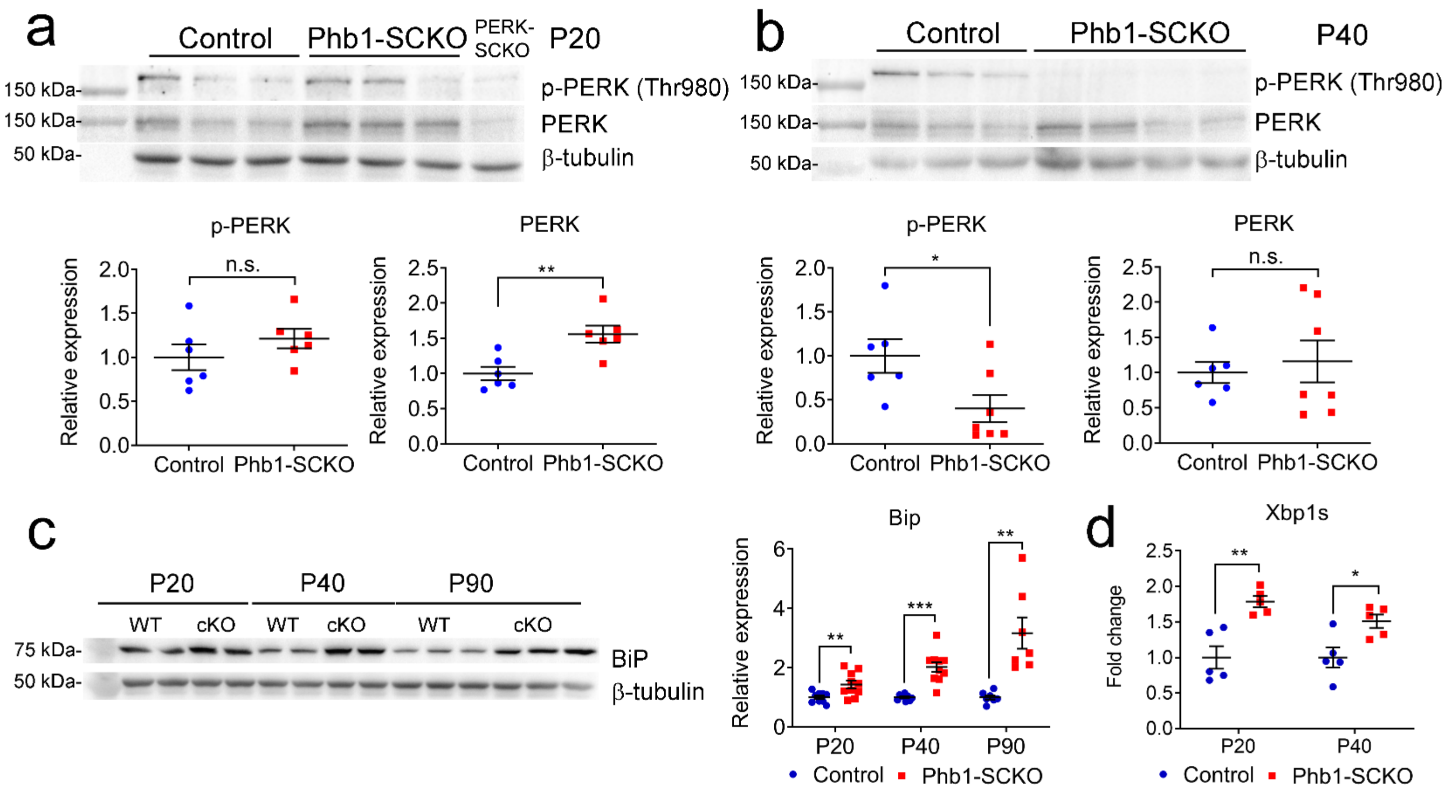

Supplementary Fig. 14. Endoplasmic reticulum (ER) stress seems to be activated as a consequence of mitochondrial stress. Deletion of *Phb1* does not cause phosphorylation of protein kinase R (PKR)-like endoplasmic reticulum kinase (PERK) at postnatal day 20 (P20) (**a**) or P40 (**b**). N = 6-7 animals per genotype. Unpaired two-tailed t-test [p-PERK P20 (t=0.2419, df=4, p=0.27), PERK P20 (t=2.738, df=4, p=0.0047), p-PERK P40 (t=2.514, df=5, 0.032), PERK P40 (t=1.563, df=5, p=0.66)]. However, there is a significant increase in the expression of the ER chaperone Binding immunoglobulin protein (BiP) by western blot (**c**). N = 7-10 animals per genotype. Unpaired two-tailed t-test [P20 (t=2.948, df=17, p=0.009), P40 (t=5.747, df=17, p=0.000024), P90 (t=4.069, df=12, p=0.0016)]. (**d**) There is also an upregulation of the alternative splicing of the X-box binding protein 1 (Xbp1) by RT-qPCR. N = 5 animals per genotype. Unpaired two-tailed t-test [P20 (t=4.434, df=8, p=0.0022), P40 (t=2.998, df=8, p=0.017)]. Data are presented as mean +/- SEM. * $p<0.05$; ** $p<0.01$; *** $p<0.001$. n.s. = non-significant.

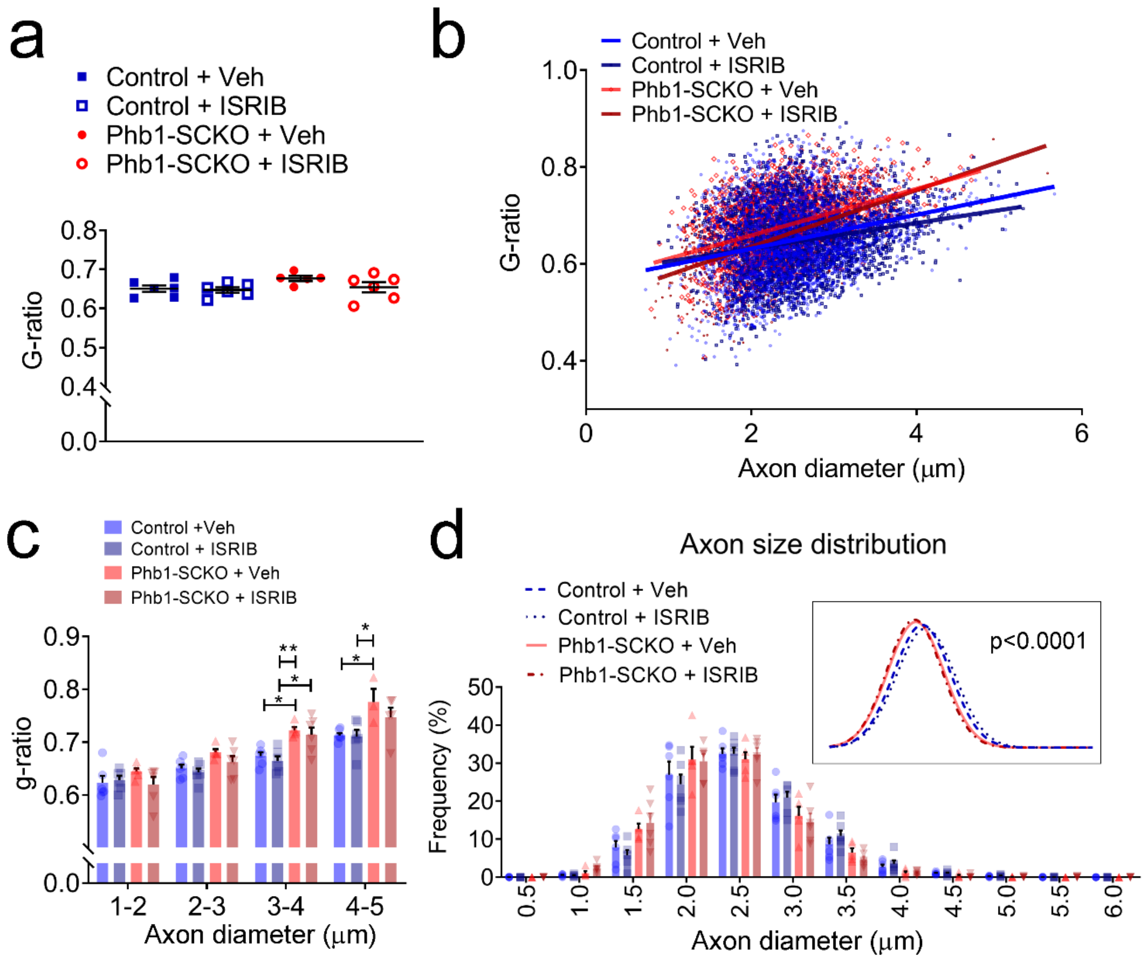

Supplementary Fig. 15. Only minor changes in the g-ratio are visible in P40 tibial nerves as a consequence of *Phb1* deletion. (**a**) There is no difference between groups in average g-ratio. N = 5-6 animals per genotype. Two-way ANOVA corrected for multiple comparisons using the Holm-Sidak method. (**b-c**) Larger caliber axons of Phb1-SCKO mice present with thinner myelin (higher g-ratio). N = 5-6 animals per genotype. Two-way ANOVA corrected for multiple comparisons using the Holm-Sidak method. F (3,73) group = 13.09, p<0.001; F (3,73) treatment = 68.45, p<0.001; $p_{3\text{-}4\text{-}Control+Veh\_Phb1\text{-}CKO+Veh}$=0.0405; $p_{3\text{-}4\text{-}Control+ISRIB\_Phb1\text{-}CKO+Veh}$=0.0069; $p_{3\text{-}4\text{-}Control+ISRIB\_Phb1\text{-}CKO+ISRIB}$= 0.0237; $p_{4\text{-}5\text{-}Control+Veh\_Phb1\text{-}CKO+Veh}$=0.016; $p_{4\text{-}5\text{-}Control+ISRIB\_Phb1\text{-}CKO+Veh}$=0.012. (**d**) Phb1-SCKO mice (regardless of treatment) show a shift towards smaller axonal size. Non-linear regression using a Gaussian curve followed by extra sum-of-squares F test [F (66,207) =17.48]. Data are presented as mean +/- SEM. * p<0.05; ** p<0.01; *** p<0.001. n.s. = non-significant.

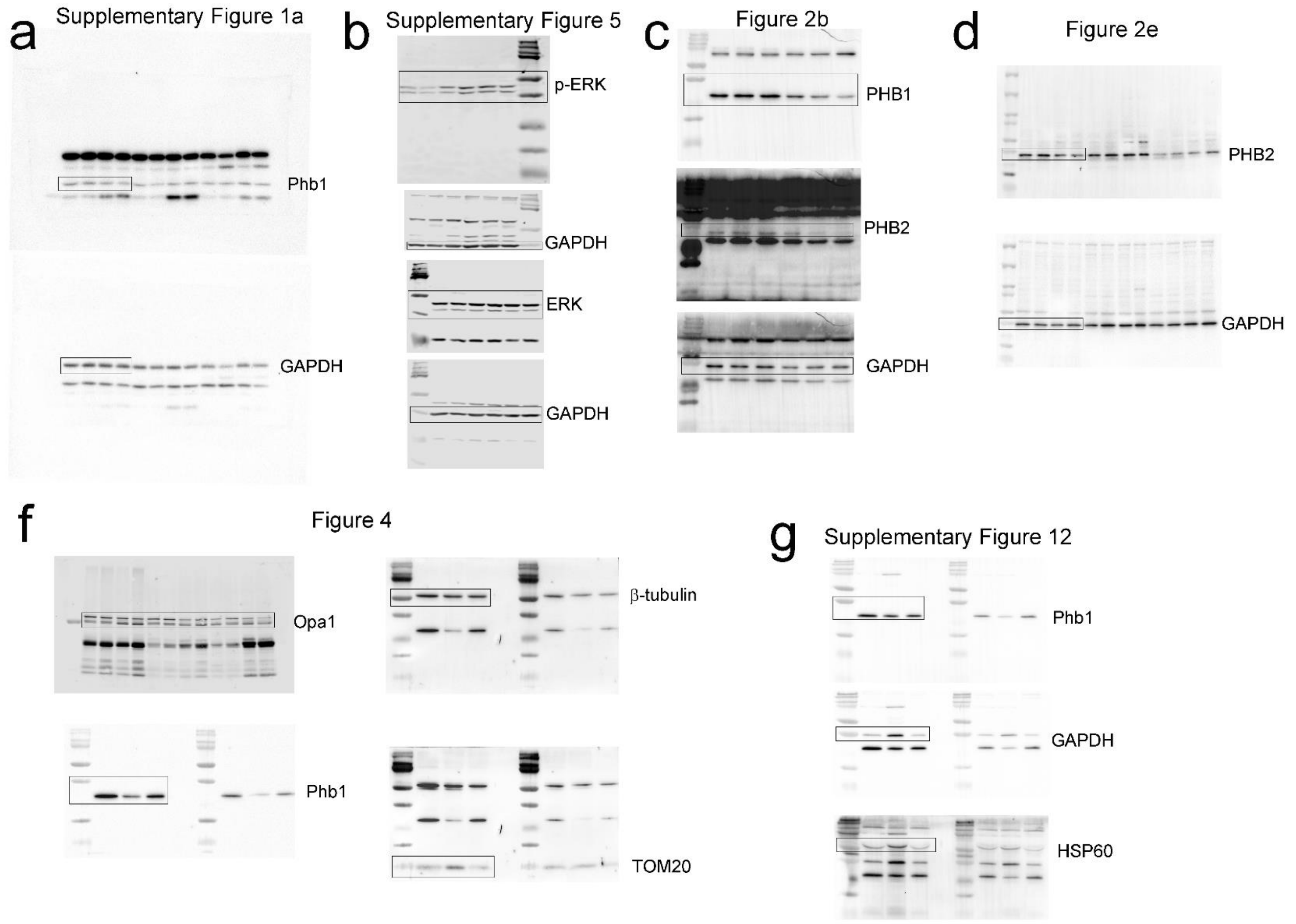

Supplementary Fig. 16. Part 1 of uncropped membranes of representative western blots in this study.

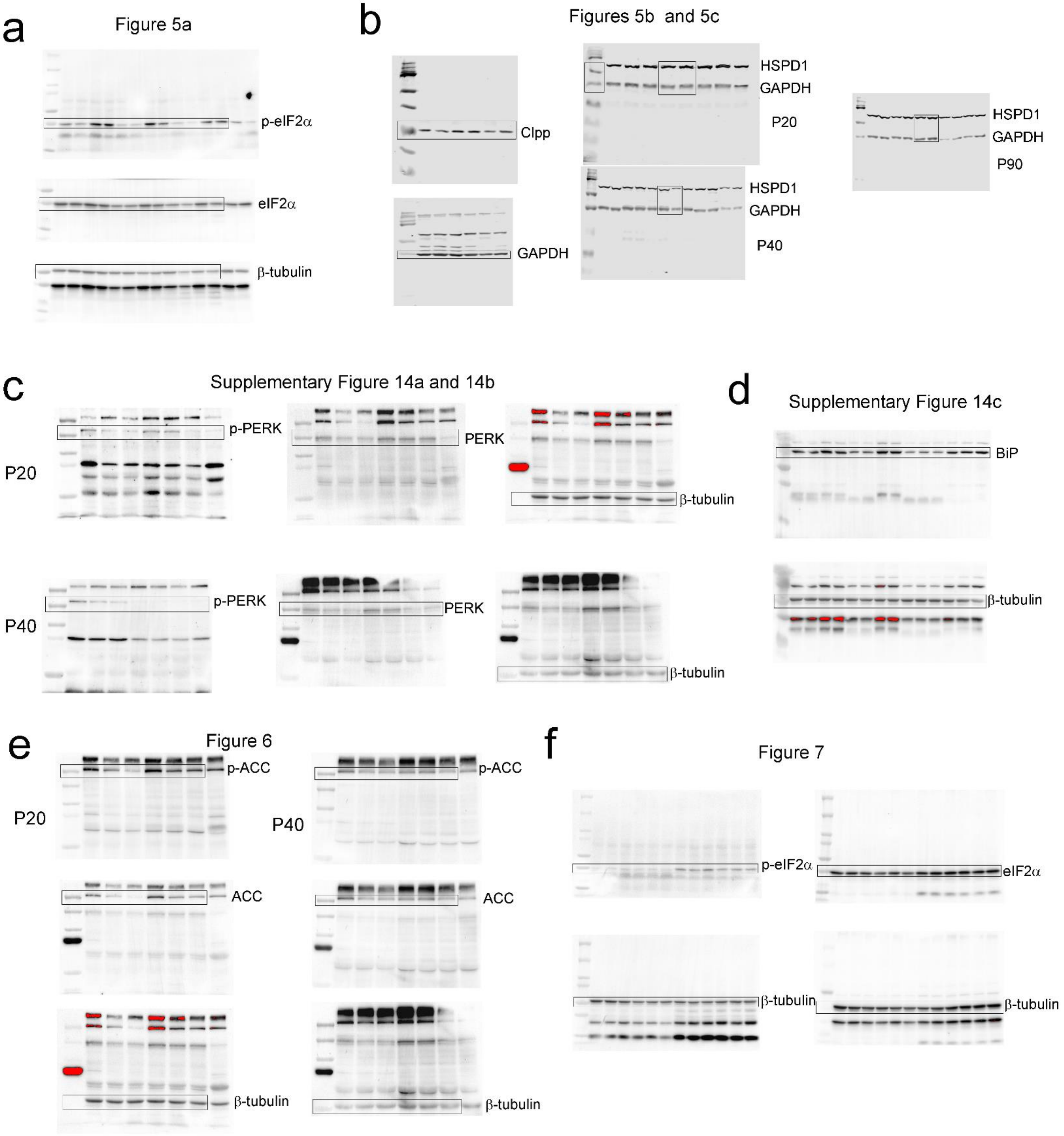

Supplementary Fig. 17. Part 2 of uncropped membranes of representative western blots in this study.

References

1 Poitelon, Y. *et al.* YAP and TAZ control peripheral myelination and the expression of laminin receptors in Schwann cells. *Nat Neurosci* **19**, 879-887, doi:10.1038/nn.4316 (2016).

2 Lazarou, M. *et al.* The ubiquitin kinase PINK1 recruits autophagy receptors to induce mitophagy. *Nature* **524**, 309-314, doi:10.1038/nature14893 (2015).

3 Viader, A. *et al.* Aberrant Schwann cell lipid metabolism linked to mitochondrial deficits leads to axon degeneration and neuropathy. *Neuron* **77**, 886-898, doi:10.1016/j.neuron.2013.01.012 (2013).

4 Ro, S. *et al.* The mitochondrial genome encodes abundant small noncoding RNAs. *Cell Research* **23**, 759-774, doi:10.1038/cr.2013.37 (2013).

5 Viader, A. *et al.* Schwann cell mitochondrial metabolism supports long-term axonal survival and peripheral nerve function. *J Neurosci* **31**, 10128-10140, doi:10.1523/jneurosci.0884-11.2011 (2011).

6 Medina, D. L. *et al.* Lysosomal calcium signalling regulates autophagy through calcineurin and TFEB. *Nature Cell Biology* **17**, 288-299, doi:10.1038/ncb3114 (2015).
