# Supplementary material for: Prohibitin 1 is essential to preserve mitochondria and myelin integrity in Schwann cells
Source: Nat Commun. 2021 Jun 2;12:3285. doi: 10.1038/s41467-021-23552-8 (PMC8172551; doi:10.1038/s41467-021-23552-8)
Supplement: Supplementary file 3 — Description of Additional Supplementary Files [file 41467_2021_23552_MOESM3_ESM.pdf]

Description of Additional Supplementary Files

File Name: Supplementary Movie 1
Description: Phenotype of Phb1-SCKO mice at P90. Note the hindlimb paresis in one of the animals as compared to its littermate control. PHB1-SCKO animal starts video bottom left

File Name: Supplementary Movie 2
Description: Video 1 with side-by-side comparison of mitochondrial dynamics in isolated SCs of Phb1-SCKO and control animals.

File Name: Supplementary Movie 3
Description: Video 2 with side-by-side comparison of mitochondrial dynamics in isolated SCs of two other Phb1-SCKO and control animals.
