# Supplementary material for: Prohibitin 1 is essential to preserve mitochondria and myelin integrity in Schwann cells
Source: Nat Commun. 2021 Jun 2;12:3285. doi: 10.1038/s41467-021-23552-8 (PMC8172551; doi:10.1038/s41467-021-23552-8)
Supplement: Supplementary file 7 — Reporting Summary [file 41467_2021_23552_MOESM7_ESM.pdf]

nature research

Corresponding author(s): M Laura Feltri

Last updated by author(s): Apr 12, 2021

Reporting Summary

Nature Research wishes to improve the reproducibility of the work that we publish. This form provides structure for consistency and transparency in reporting. For further information on Nature Research policies, see our Editorial Policies and the Editorial Policy Checklist.

Statistics

For all statistical analyses, confirm that the following items are present in the figure legend, table legend, main text, or Methods section.

n/a	Confirmed	
☐	☒	The exact sample size (n) for each experimental group/condition, given as a discrete number and unit of measurement
☐	☒	A statement on whether measurements were taken from distinct samples or whether the same sample was measured repeatedly
☐	☒	The statistical test(s) used AND whether they are one- or two-sided<br>*Only common tests should be described solely by name; describe more complex techniques in the Methods section.*
☐	☒	A description of all covariates tested
☐	☒	A description of any assumptions or corrections, such as tests of normality and adjustment for multiple comparisons
☐	☒	A full description of the statistical parameters including central tendency (e.g. means) or other basic estimates (e.g. regression coefficient) AND variation (e.g. standard deviation) or associated estimates of uncertainty (e.g. confidence intervals)
☐	☒	For null hypothesis testing, the test statistic (e.g. F, t, r) with confidence intervals, effect sizes, degrees of freedom and P value noted<br>*Give P values as exact values whenever suitable.*
☒	☐	For Bayesian analysis, information on the choice of priors and Markov chain Monte Carlo settings
☒	☐	For hierarchical and complex designs, identification of the appropriate level for tests and full reporting of outcomes
☒	☐	Estimates of effect sizes (e.g. Cohen's d, Pearson's r), indicating how they were calculated

Our web collection on statistics for biologists contains articles on many of the points above.

Software and code

Policy information about availability of computer code

Data collection	Images from semithin sections were aquired using the Q-Capture Pro V7.0.4324.5 software (QImaging, Inc.). Confocal images were acquired using the Leica SP5II microscope running the LAS AF 2.7.9723.3 software (Leica). The remaining fluorescence images were obtained with a Zeiss ApoTome microscope (Zeiss Observer.Z1 AX10) running the AxioVision 4.8.2.0 software (Zeiss). Western blot images were acquired using either the Image lab 6.0 software (Biorad) for blots imaged with the ChemiDoc XRS or in the Image Studio Lite 5.2 (Odyssey) for blots imaged with the Odyssey CLx.
Data analysis	Leica QWin software (V 3.5.1) was used to analyze the g-ratios. PTGui software v.10 (New House Internet Services BV) was used to reconstruct a complete image of the nerve. ImageJ Fiji v1.52p was used for analysis of microscope images. GraphPad Prism 6.01 was used for the statistical analyses.<br>Image J macros were used to run some analyses and are described in the methods. These are available with no restriction under request.<br>The image J macro used to quantify parameters of the mitochondrial network in PhAM mice is available at https://github.com/gdfnunes/3D-analysis-mitochondria.

For manuscripts utilizing custom algorithms or software that are central to the research but not yet described in published literature, software must be made available to editors and reviewers. We strongly encourage code deposition in a community repository (e.g. GitHub). See the Nature Research guidelines for submitting code & software for further information.

Data

Policy information about availability of data

All manuscripts must include a data availability statement. This statement should provide the following information, where applicable:

- Accession codes, unique identifiers, or web links for publicly available datasets
- A list of figures that have associated raw data
- A description of any restrictions on data availability

The data supporting the findings of this study are available within the article and its supplementary information files. All raw data used in figures and the output of the statistical tests are contained in the Source data file 1. All original data and biological resources (mouse strains, plasmids, etc.) are available from the corresponding author upon reasonable request.

Field-specific reporting

Please select the one below that is the best fit for your research. If you are not sure, read the appropriate sections before making your selection.

[x] Life sciences [ ] Behavioural & social sciences [ ] Ecological, evolutionary & environmental sciences

For a reference copy of the document with all sections, see nature.com/documents/nr-reporting-summary-flat.pdf

Life sciences study design

All studies must disclose on these points even when the disclosure is negative.

Sample size	No power analysis was performed, but our sample sizes are similar to those generally used in the field and in line with projects our laboratory carried out in the past: For analysis of morphology and IHC, a minimum sample size of 3 is commonly used, while analyses of biochemistry, behavior, nerve conduction and molecular biology usually use a N of at least 4-5:<br>- Weinstock, N. I. et al. Macrophages Expressing GALC Improve Peripheral Krabbe Disease by a Mechanism Independent of Cross-Correction. Neuron 107, 65-81.e69, doi:10.1016/j.neuron.2020.03.031 (2020).<br>- Poitelon, Y. et al. YAP and TAZ control peripheral myelination and the expression of laminin receptors in Schwann cells. Nat Neurosci 19, 879-887, doi:10.1038/nn.4316 (2016).<br>- Babetto, E., Wong, K. & Beirowski, B. A glycolytic shift in Schwann cells supports injured axons. Nature neuroscience 23, doi:10.1038/s41593-020-0689-4 (2020).<br>- Eichel, M. A. et al. CMTM6 expressed on the adaxonal Schwann cell surface restricts axonal diameters in peripheral nerves. Nature Communications 11, doi:10.1038/s41467-020-18172-7 (2020).<br>- Fröb, F. et al. Ep400 deficiency in Schwann cells causes persistent expression of early developmental regulators and peripheral neuropathy. Nature Communications 10, doi:10.1038/s41467-019-10287-w (2019).
Data exclusions	No data were excluded from the analyses.
Replication	All the data reported within this manuscript has been replicated in our lab, and all replication efforts were successful. Analyses were performed with at least three biological replicates. Whenever possible, each experiment was also divided into a smaller group and performed independently on different days, confirming the consistency of our results.
Randomization	Allocation of animals to ISRIB or vehicle treated-groups was randomized.
Blinding	Data collection and analysis were performed blind to the conditions of the experiments and genotype of the mice in the following experiments: quantifications derived from semithin, EM and IHC experiments; analyses of behavior and nerve conduction; and analyses of mitochondrial morphology, dynamics, potential and mitophagy. However, due to the severity of the phenotype, it was not possible to completely prevent investigators from identifying if the animal was WT or mutant in some analyses.

Reporting for specific materials, systems and methods

We require information from authors about some types of materials, experimental systems and methods used in many studies. Here, indicate whether each material, system or method listed is relevant to your study. If you are not sure if a list item applies to your research, read the appropriate section before selecting a response.

Materials & experimental systems

n/a	Involved in the study	
☐	☒	Antibodies
☒	☐	Eukaryotic cell lines
☒	☐	Palaeontology and archaeology
☐	☒	Animals and other organisms
☒	☐	Human research participants
☒	☐	Clinical data
☒	☐	Dual use research of concern

Methods

n/a	Involved in the study	
☒	☐	ChIP-seq
☒	☐	Flow cytometry
☒	☐	MRI-based neuroimaging

Antibodies

Antibodies used

KDEL (Thermo Fisher Scientific #PA1-013), Kv1.1 (Alomone #APC-009), p75NTR (Cell signaling #8238), S100b (Daki #ZO311), Neurofascin (R&D Systems #AF3235), TOM20 (Proteintech #11802-1-AP), HSPD1 (Proteintech # 15282-1-AP), P0 (Aves #PZO0308), MBP (Smi99) (Biolegend #808401), p-H3 (Millipore #06-576), Ki-67 (Thermo Fisher Scientific #14-5698-80), SOX10 (Cell signaling #89356), PHB1 (Abcam #ab28172), Opa1 (BD Biosciences # 612606), Erk1/2 (Cell signaling #9102), p-ERK1/2 (Cell signaling #9101), b-tubulin (Novus Biologicals #NB600-936), TOM20 (BD Biosciences #612278), GAPDH (Sigma #G9545), eIF2a (Cell signaling #5324), p-eIF2a (Cell signaling #3398), Clpp (Proteintech #15698-1-AP), PERK (Cell signaling #3192), p-PERK (Cell signaling #3179), Bip (Novus Biologicals #NB300-520), ACC (Cell signaling #3662), p-ACC (Cell signaling #3661), PHB2 (Millipore # ab10198), Alexa 488 donkey anti-rabbit IgG 1/1000 (Jackson ImmunoResearch #711-545-152), Cy3 donkey anti-chicken IgY 1/500 (Jackson ImmunoResearch #703-165-155), Cy3 donkey anti-mouse IgG 1/500 (Jackson ImmunoResearch #715-165-150), rhodamine (TRITC) anti-rabbit IgG 1/500 (Jackson ImmunoResearch #711-025-152), Alexa 594-Streptavidin secondary antibody (Jackson ImmunoResearch #016-580-084).

Validation

The manuscript provides validation on the following antibodies using conditional-KO mice: PERK (Cell signaling #3192), p-PERK (Cell signaling #3179);
The following antibody was validated in the manuscript using shRNA: Phb1 (Abcam #ab28172);
All the other antibodies used in this study have been validated by the manufacturers and validation was supported by previous publications. We also confirmed that all the primary antibodies used in this study resulted in staining patterns compatible with the expected cellular localization and/or WB bands of the expected MW. Below we list relevant publications and validation statements available from websites of commercial antibodies:
KDEL (Thermo Fisher Scientific #PA1-013): Giese A. P. J. et al., Nat Commun. 2017 Jun 29;8(1):43 AND "PA1-013 detects KDEL from human, rat, mouse and hamster.PA1-013 has been successfully used in Western blot, immunofluorescence and immunoprecipitation procedures."
Kv1.1 (Alomone #APC-009):Poitelon Y. et al., J Neurochem. 2018 May;145(3):245-257. "Anti-KV1.1 (KCNA1) Antibody (#APC-009) is a highly specific antibody directed against an epitope of the mouse protein. The antibody can be used in western blot, immunohistochemistry, immunocytochemistry, and immunoprecipitation applications."
p75NTR (Cell signaling #8238): Sapkota D. et al., Neural Dev. 2020 Feb 20;15(1):2. AND "p75NTR (D4B3) XP® Rabbit mAb recognizes endogenous levels of total p75NTR protein. Species Reactivity: Human, Mouse, Rat".
S100b (Dako #ZO311): Trias E. et al., Glia. 2020 Jun;68(6):1165-1181.
Neurofascin (R&D Systems #AF3235): Susuki K. et al., J Neurosci. 2018 Jul 4;38(27):6063-6075 AND "Detects human, mouse, rat Neurofascin in Western blots and rat Neurofascin in direct ELISAs."
TOM20 (Proteintech #11802-1-AP): Baranov et a., S. V. et al., Proc Natl Acad Sci U S A
. 2019 Jan 8;116(2):650-659. AND "11802-1-AP targets TOM20 in WB, IP, IHC, IF, FC, ELISA applications and shows reactivity with human, mouse, rat samples."
HSPD1 (Proteintech # 15282-1-AP): Flippo K. H. et al., J Neurosci. 2018 Sep 19;38(38):8233-8242. AND "15282-1-AP targets HSP60 in WB, IP, IHC, IF, ELISA applications and shows reactivity with human, mouse, rat samples."
P0 (Aves #PZO0308): Weinstock N. I. et al., Neuron. 2020 Jul 8;107(1):65-81.e9.
MBP (Smi99) (Biolegend #808401): Reed C. B. et al., J Neurosci. 2020 Aug 5;40(32):6165-6176. AND "The antibody detects myelin basic protein from most mammalian species."
Phb1 (Abcam #ab28172): Poitelon Y et al., Nat Commun. 2015 Sep 18;6:8303.
Opa1 (BD Biosciences # 612606): Pereira R. O. et al., EMBO J. 2017 Jul 14;36(14):2126-2145 AND "This antibody is routinely tested by western blot analysis."
Erk1/2 (Cell signaling #9102): Poitelon Y et al., Nat Commun. 2015 Sep 18;6:8303.
p-ERK1/2 (Cell signaling #9101): Poitelon Y et al., Nat Commun. 2015 Sep 18;6:8303.
b-tubulin (Novus Biologicals #NB600-936): Weinstock N. I. et al., Neuron. 2020 Jul 8;107(1):65-81.e9.
TOM20 (BD Biosciences #612278): Gillingham A. K. et al., Elife. 2019 Jul 11;8:e45916. doi: 10.7554/eLife.45916.
GAPDH (Sigma #G9545): Belin S. et al., Hum Mol Genet. 2019 Apr 15;28(8):1260-1273.
eIF2a (Cell signaling #5324): Aggarwal et al., J Biol Chem. 2019 Mar 1;294(9):3152-3168 AND "eIF2α (D7D3) XP® Rabbit mAb detects endogenous levels of total eIF2α protein. Species Reactivity: Human, Mouse, Rat, Monkey"
p-eIF2a (Cell signaling #3398): Zhu et al., Nat Commun. 2019 Mar 6;10(1):1084. AND "Phospho-eIF2α (Ser51) (D9G8) XP® Rabbit mAb detects endogenous eIF2α only when phosphorylated at Ser51. The antibody does not recognize eIF2α phosphorylated at other sites. Species Reactivity: Human, Mouse, Rat, Monkey, D. melanogaster"
Clpp (Proteintech #15698-1-AP): Richter U et al., J Cell Biol. 2015 Oct 26;211(2):373-89. AND "15698-1-AP targets CLPP in WB, IP, IHC, IF, ELISA applications and shows reactivity with human, mouse, rat samples."
PERK (Cell signaling #3192): Logue S. E. et al., Nat Commun. 2018 Aug 15;9(1):3267. AND "PERK (C33E10) Rabbit mAb detects endogenous levels of total PERK protein. Species Reactivity: Human, Mouse, Rat, Monkey"

p-PERK (Cell signaling #3179): Jo S. et al., J Clin Invest. 2019 Jan 2;129(1):230-245. AND "Phospho-PERK (Thr980) (16F8) Rabbit mAb detects endogenous levels of PERK phosphorylated at Thr980. Species predicted to react based on 100% sequence homology: Mouse"
Bip (Novus Biologicals #NB300-520): Sidoli M. et al., J Neurosci. 2016 Nov 2;36(44):11350-11361.
ACC (Cell signaling #3662): Viader A. et al., Neuron. 2013 Mar 6;77(5):886-98.
p-ACC (Cell signaling #3661): Viader A. et al., Neuron. 2013 Mar 6;77(5):886-98.
PHB2 (Millipore # ab10198): Poitelon Y et al., Nat Commun. 2015 Sep 18;6:8303.
p-H3 (Millipore #06-576): Poitelon Y et al., Nat Commun. 2015 Sep 18;6:8303.
Ki-67 (Thermo Fisher Scientific #14-5698-80): Reed C. B. et al., J Neurosci. 2020 Aug 5;40(32):6165-6176.
SOX10 (Cell signaling #89356): Reed C. B. et al., J Neurosci. 2020 Aug 5;40(32):6165-6176.

Animals and other organisms

Policy information about studies involving animals; ARRIVE guidelines recommended for reporting animal research

Laboratory animals	Mpz-Cre and Phb1 floxed animals were previously described (Feltri et al. 1999 and He B. et al. 2011, respectively). Mice were also crossed to Thy1-YFP (Ey, B. et al. 2007) and PhAM (Jackson laboratories Stock No: 018397) reporter lines. Animals were kept in a C57BL/6 and 129 mixed genetic background and analyses were performed from littermates. Both male and female mice were used in the study and number of animals of each sex in each experiment was matched whenever possible. Age of the animals analyzed in this study is reported in each figure, but varied from postnatal day 10 (P10) to P120.
Wild animals	No wild animals have been used in this study
Field-collected samples	No field-collected samples have been used in this study
Ethics oversight	All animal experiments were approved by the Institutional Animal Care and Use Committee (IACUC) of the Roswell Park Cancer Institute and the regulatory authorities at the University at Buffalo.

Note that full information on the approval of the study protocol must also be provided in the manuscript.
